# Three-Dimensional Evaluation of the Upper Airway Morphological Changes in Growing Patients with Skeletal Class III Malocclusion Treated by Protraction Headgear and Rapid Palatal Expansion: A Comparative Research

**DOI:** 10.1371/journal.pone.0135273

**Published:** 2015-08-07

**Authors:** Xueling Chen, Dongxu Liu, Ju Liu, Zizhong Wu, Yongtao Xie, Liang Li, Hong Liu, Tiantian Guo, Chen Chen, Shijie Zhang

**Affiliations:** 1 Department of Orthodontics, School of Dentistry, Shandong University, Jinan, China; 2 Department of Orthodontics, Shandong Provincial Key Laboratory of Oral Biomedicine, School of Dentistry, Shandong University, Jinan, China; 3 Department of Stomatology, Qilu Hospital of Shandong University, Jinan, China; 4 Medical Research Center, Shandong Provincial Qianfoshan Hospital, Shandong University, Jinan, China; 5 Department of Stomatology, The Chinese People’s Liberation Army 88 Hospital, Taian, China; 6 Department of Stomatology, Traditional Chinese Medical Hospital of Shandong Province, Jinan, China; Tufts University, UNITED STATES

## Abstract

**Objective:**

The aim of this study was to evaluate the morphological changes of upper airway after protraction headgear and rapid maxillary expansion (PE) treatment in growing patients with Class III malocclusion and maxillary skeletal deficiency compared with untreated Class III patients by cone-beam computed tomography (CBCT).

**Methods:**

Thirty growing patients who have completed PE therapy were included in PE group. The control group (n = 30) was selected from the growing untreated patients with the same diagnosis. The CBCT scans of the pre-treatment (T1) and post-treatment (T2) of PE group and the control group were collected. Reconstruction and registration of the 3D models of T1 and T2 were completed. By comparing the data obtained from T1, T2 and control group, the morphological changes of the upper airway during the PE treatment were evaluated.

**Results:**

Comparing with the data from T1 group, the subspinale (A) of maxilla and the upper incisor (UI) of the T2 group were moved in the anterior direction. The gnathion (Gn) of mandible was moved in the posterior-inferior direction. The displacement of the hyoid bone as well as the length and width of dental arch showed significant difference. The volume and mean cross-sectional area of nasopharynx, velopharynx and glossopharynx region showed significant difference. The largest anteroposterior/the largest lateral (AP/LR) ratios of the velopharynx and glossopharynx were increased, but the AP/LR ratio of the hypopharynx was decreased. In addition, the length and width of the maxillary dental arch, the displacement of the hyoid bone, the volume of nasopharynx and velopharynx, and the AP/LR ratio of the hypopharynx and velopharynx showed significant difference between the data from control and T2 group.

**Conclusion:**

The PE treatment of Class Ⅲ malocclusion with maxillary skeletal hypoplasia leads to a significant increase in the volume of nasopharynx and velopharynx.

## Introduction

Class III malocclusion is characterized by a deficiency of the maxilla, or prognathism of the mandible, or the maxilla and mandible dysplasia [1]. Patients with this type of malocclusion commonly present a concave profile, an anterior crossbite relationship, and a Class III molar relationship [2]. Numerous studies demonstrated that maxillary or mandibular abnormalities change the volume of the oral cavity, and affect the morphology of the upper airway [3].The untreated Class III malocclusion patients with the craniofacial anomalies usually have the constriction of velopharynx and nasal cavity, nasal obstruction or choanal stenosis, which is caused by the severe maxillary hypoplasia [4,5]. On the other hand, the untreated Class III malocclusion patients have significantly larger oropharynx compared with the Class I malocclusion [6]. The PE treatment leads to forward displacement and growth of the maxilla, meanwhile it limits mandible growth and promotes mandibular clockwise rotation. Therefore, a detailed assessment of the changes in upper airway morphology in skeletal Class Ⅲ malocclusion patients is essential for the routine orthodontic planning and treatment.

Previous studies evaluated the changes of the upper airway after the protraction headgear treatment and/or rapid maxillary expansion treatment using 2D method or using 3D method without control group [7–11]. Using the cephalometric radiographs of the Class III malocclusion patients, the effects of protraction headgear on the upper airway were assessed, which indicated that the maxillary protraction produces the change of the size of the upper airway [7,8]. Another study found that the changes of upper airway at the upper pharyngeal after rapid maxillary expansion [9]. In addition, analysis of the cephalometric radiographs of maxillary protraction patients suggested that maxillary protraction with or without expansion fails to produce a significant increase in upper airway dimensions at the short term [10]. Most of the previous studies evaluated the changes of the upper airway using cephalometric radiography. This method limits the accuracy of the measurements of upper airway since the two-dimensional image only shows anteroposterior changes in sagittal plane and fails to provide a full-scale view of the upper airway [11].Therefore, 3D evaluation of the upper airway in growing patients during PE treatment needs to be established.

CBCT emerged an acceptable technique for evaluation of upper airway morphology due to its lower-radiation and greater spatial resolution [12,13,14,15]. By identifying the stable structures in the cranial base, it is possible to perform registration of pre- and post treatment 3D models. Thus the 3D registration method may help us assess the upper airway changes accurately [16].

The aim of this study was to evaluate the changes of upper airway by 3D registration for the growing patients of skeletal Class III malocclusion with maxillary skeletal deficiency during PE treatment. As all patients are in active growth period, their normal growth may affect the morphology of the upper airway during the PE treatment period. Thus, we set up an untreated control group in this study. The control group was selected from the patients with the same diagnosis as the PE group. The age, gender and growth conditions of the control group are well-matched with patients of PE group at T2.

## Materials and Methods

### Ethics statement

The study was approved by the Research Ethic Committee of Shandong University Dental School (protocol number 2014068, Jinan, China). The parents or guardians of all the subjects received information about the procedure including the possible damage from CBCT radiation and signed the written informed consent form. The individual in this manuscript has given written informed consent (as outlined in PLOS consent form) to publish these case details.

### Subject

The CBCT records of 60 patients (28 girls and 32 boys) were collected from the Department of Orthodontics, School of Dentistry, Shandong University and Department of Stomatology, Qilu Hospital of Shandong University during 2012–2014. All the patients were in the growth period with Class III malocclusion and maxillary skeletal deficiency. The PE group included 30 growing patients who had completed PE treatment (14 girls and 16 boys). The mean age of the T1 group was 9.56±0.22 years. The mean age of the T2 group was 10.32±0.33 years. The control group (14 girls and 16 boys, mean age 10.41±0.42 years) was selected to match with PE group by the age, gender and growth condition. The growth conditions include height, bone age, body weight and BMI.

All the patients were selected based on the following criteria:

1. At a prepubertal stage of skeletal maturity. The skeletal maturation age was assessed using their hand-wrist radiographs and the vertebral maturation method [17].
2. Class III molar relationship and anterior crossbite in late mixed or early permanent dentition.
3. Skeletal Class III malocclusion with maxillary deficiency. The ANB angle was between 0° and -4°, SNA <78.8°, A-NP<0 mm, Wits appraisal was -2 mm or less[10].
4. No mandibular forward functional displacement.
5. No other congenital anomalies, facial neoplasms, potential airway abnormalities or previous orthodontic treatment.

### Protraction headgear combined with rapid expansion

**Fig 1:**
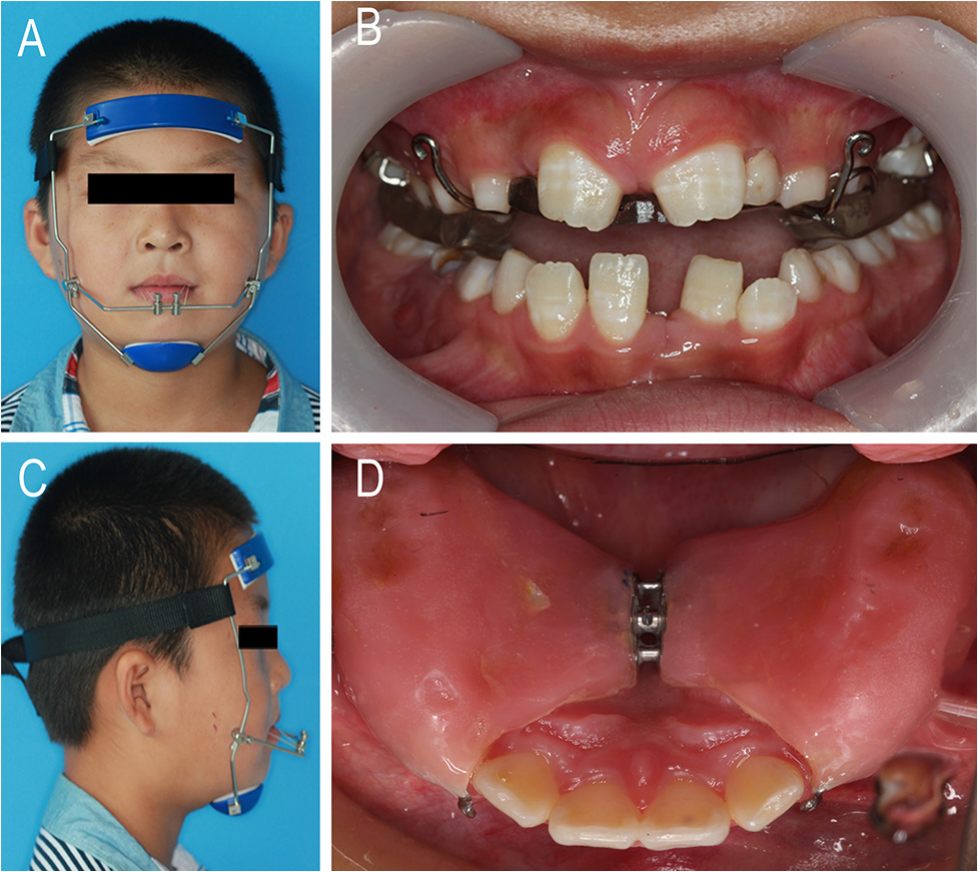
The patients of the PE group. A) The facemask of the protraction appliance. B) The elastics were oriented in a downward and forward direction at an angle of approximately 30° relative to the occlusal plane.C) The protraction appliance hooks in the oral cavity. D)The oral appliance of the rapid palate expansion.

Thirty patients were treated by the PE appliances, a combination of maxillary protraction headgear and expansion device. An acrylic cap splint Hyrax was used as the expansion device. The screw of the rapid expansion appliance was activated twice a day for a period of 2 or 3 weeks. After the maxillary expansion, the screw of the expansion appliance was blocked by acrylic. Maxillary protraction headgear was performed using a facemask (Xinya dentistry material company, Hangzhou, China).The elastics were oriented in a forward and downward direction at an angle of approximately 30° relative to the occlusal plane. The forces of 500g on each side were used during the treatment (Fig 1). The patients were instructed to wear the appliance for at least 14 hours per day. The mean treatment duration was 7±1.21 months. The patients were examined after every 3 weeks until the treatment was completed. The PE appliance was removed when an overjet of 3–5 mm, and Class I orⅡ relationship of molar and canine were achieved [8,18].

### CBCT Data Acquisition

CBCT scans were performed for all the patients in control and PE groups by using the CBCT scanner (KaVo Dental GmbH, Bismarckring, Germany), which is 0.30 voxel resolution with the scanning parameter of 5 mA, 120 kV. The slice thickness was 0.4 mm, and the scan time was 8.9 seconds.

The CBCT scans of control group were collected before orthodontic treatment. In the PE group, the CBCT data of T1 and T2 were collected before and after the PE treatment, respectively. During CBCT scanning, all the patients were instructed to maintain an upright standing posture and natural head position. The patients’ Frankfort planes were adjusted parallel to the ground. All the patients were instructed to keep awake with the maximum intercuspation during the CBCT scanning. They maintained the rest position of the tongue, which was in contact with anterior hard palate without touching the anterior teeth. All the CBCT data were exported in the Digital Imaging and Communications in Medicine (DICOM) format [19,20].

### 3D Virtual Model Reconstruction

All the CBCT data collected for this study were transferred into MIMICS 16.0 (Materialism’s Interactive Medical Image Control System) software. The bone tissues and upper airway morphology structure were separated by the threshold based on Hounsfield Units (HU). The lower limit of 322 HU and upper limit of 3070 HU were used for the hard tissue, while the lower limit of -1024 HU and higher limit of -368 HU were used for upper airway. Then the 3D models of both the upper airway and bone structures were constructed. All the 3D models were exported as stereolithography (STL).

### Registration of the Pre- and Post-treatment 3D Models

In MIMICS, the T1 STLs were registered with the T2 STLs models by point-registration based on the structures of anterior cranial base [19,21]. To accomplish the point registration, landmark points representing the same anatomic sites were placed on the T1 and T2 STL images [22]. MIMICS calculated the best transformation matrix according to the landmark points, and the STL model was then converted to the aim position. STL registration was accomplished by placing STL model on CBCT masks to improve the accuracy after the point registration.

### 3D measurement

**Fig 2:**
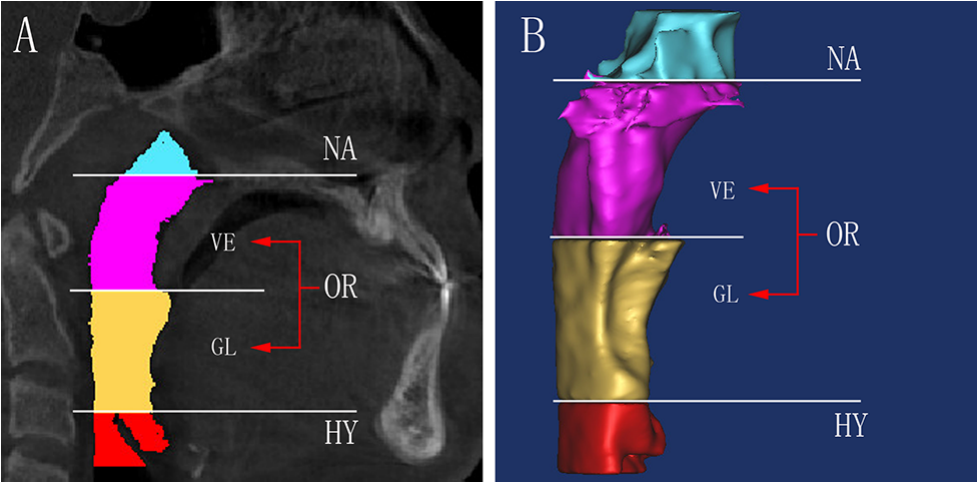
The region of the upper airway. A) The upper airway divided into 3 parts, among the oropharynx region divided into 2 parts, different region was highlighted in different colors. B) The 3D model of each region was reconstructed, the oropharynx divide into velopharynx and glossopharynx.

In this study, the upper airway was divided into 3 parts [19,23,24]: the nasopharynx (NA, the top of the upper airway to hard palate plane), oropharynx (OR, the hard palate plane to the superior border of the epiglottis) and hypopharynx (HY, the superior border of the epiglottic to the bottom of the epiglottic) by the corresponding cross-sectional slices in the midsagittal plane. The oropharynx was divided into 2 parts: the velopharynx (VE, the hard palate plane to the point of the uvula) and the glossopharynx(GL, the point of the uvula to the superior border of the epiglottic) (Fig 2).

**Fig 3:**
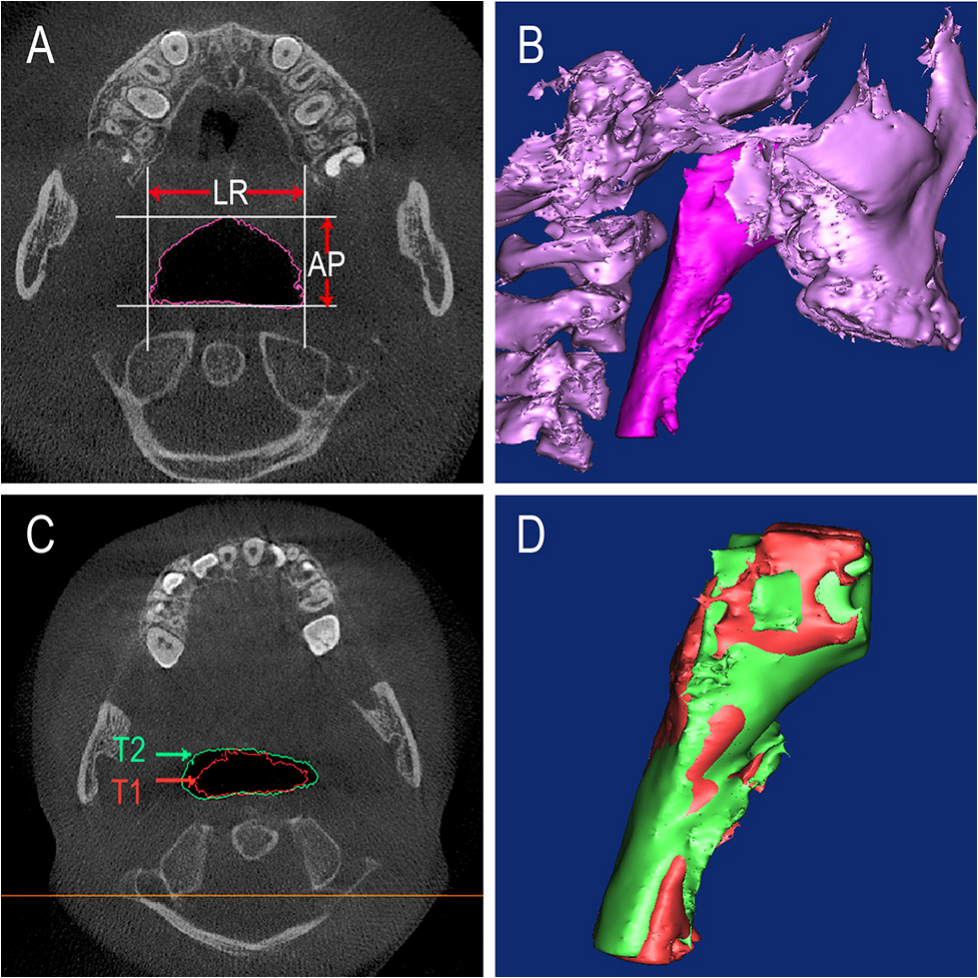
The changes of the upper airway. A) The largest lateral (LR) and anteroposterior (AP) dimensions of the airway. B) The 3D model of the upper airway and maxillary in the control subjects. C) The changes of the upper airway between T1 and T2 data in theaxial view. D) After PE treatment the 3D model of the upper airway have changed.

After reconstruction of the airway, the volume (V) and the height (h) were automatically calculated by the MIMICS software. The mean cross-sectional area (mCSA) of each region was computed as the ratio of V/h. In the axial section, the largest lateral and anteroposterior dimensions for each cross-sectional slice were measured. The AP/LR ratio was calculated to evaluate the shape of the upper airway. The cross section turns more circular when the ratio increases, while it turns more elliptic if the ratio decreases. After the registration, the morphological changes of each region were evaluated in sagittal slices and 3D models (Fig 3).

**Fig 4:**
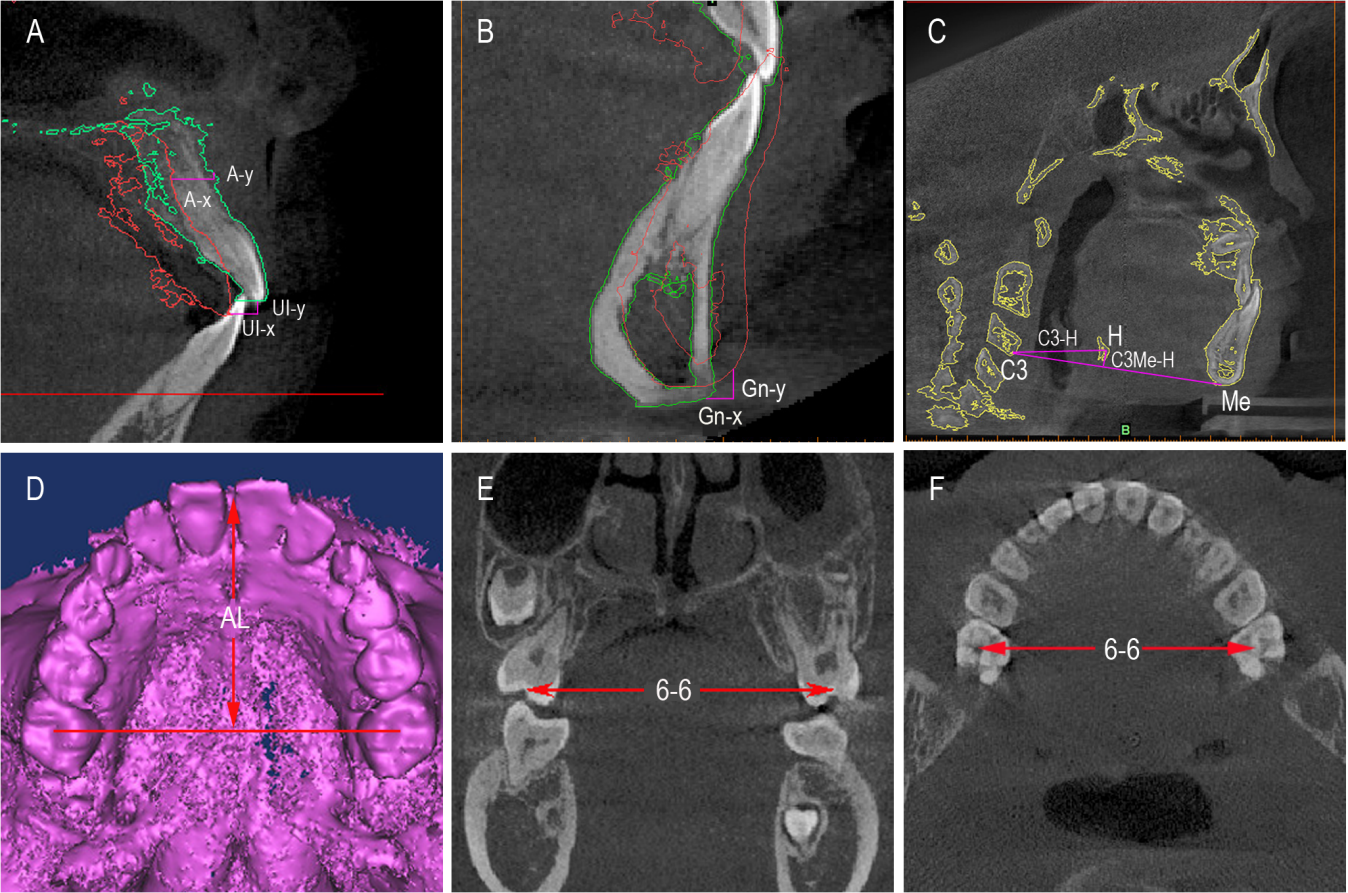
The changes of the hard tissue. A) The movement of the A point and UI point. The red contour line represent the T1 data and the green contour line represent the T2 data. B) The displacement of the mandible was assessed in horizontal (Gn-x) and vertical (Gn-y) directions. C) The horizontal position of hyoid bone was evaluated from C3 point to hyoid point (C3-H) in the midsagittal plane, and its vertical displacement was measured through the perpendicular distance between C3-menton line and H (HC3Me). D) The length of the dental arch. E) The width of the maxillary posterior arch in the coronal plane. F) The width of the maxillary posterior arch in the axial view.

In the PE group, the changes of the maxilla were evaluated in horizontal (A-x) and vertical (A-y) directions at the mid-sagittal plane. The position of upper incisor was assessed in horizontal (UI-x) and vertical (UI-y) directions. The displacement of the mandible was assessed in horizontal (Gn-x) and vertical (Gn-y) directions [21]. In the control and PE groups, the length and width of the dental arch were measured. The width of dental arch was performed as the distance between upper canine to canine (U3-U3), upper second deciduous molar to the second deciduous molar (UⅤ-UⅤ), and the first molar to the first molar (U6-U6). The horizontal position of hyoid bone was evaluated from C3 point to hyoid (H) point (C3-H) in the midsagittal plane, and its vertical displacement was measured through the perpendicular distance between C3-menton line and H point (C3Me-H) [21,25,26] (Fig 4). The definitions of landmark and measurement variables were listed in Table 1 and Table 2. Measurements were repeated three times, and the average value was calculated.

**Table 1:** Definitions of landmarks.

Symbol	Definition
Gn (gnathion)	The midpoint between the most anterior and inferior points on the outline of the mandibular symphysis
A(subspinale)	The most concave point on the bone between the anterior nasal spine point and superior prosthion point.
UI (upper incisor)	The most anterior point on the incisal edge of the upper incisor
H (hyoid)	The most anterior and inferior point of the hyoid bone
C3 (third cervical vertebra)	The anterior and inferior point of the third cervical vertebra
Me (menton)	The most inferior point on the outline of the mandibular symphysis
CSA(cross-sectional area)	The cross-sectional area of the upper airway on each CBCT sagittal plane

**Table 2:** Definitions of measurements variables.

Symbol	Description	Definition
V	Volume	The volume of each region of the airway
mCSA	Mean cross-sectional area	Mean cross-sectional area of each region of airway
LR	Left-right diameter	The lateral dimension of each region of airway on CBCT axial plane
AP	Anteroposterior dimension	The anteroposterior dimension of each region of airway on CBCT axial plane
AP/LR	Ratio of AP/LR	Ratio of AP to LR dimensions
C3-H	The third cervical vertebra to hyoid bone	The distance from C3 to H in the sagittal plane
C3Me-H	The C3-Me line to hyoid bone	The perpendicular distance between H and C3-Me line
Gn-x	The mandible advancement in the horizontal direction	The mean amount of mandible advancement at Gn in the horizontal direction
Gn-y	The mandible advancement in the vertical direction	The mean amount of mandible advancement at Gn in the vertical direction
A-x	The maxillary advancement in the horizontal direction	The mean amount of maxillary bone advancement at A in the horizontal direction
A-y	The maxillary advancement in the vertical direction	The mean amount of maxillary bone advancement at A in the vertical direction
UI-x	The upper incisor advancement in the horizontal direction	The mean amount of upper incisor advancement in the horizontal direction
UI-y	The upper incisor advancement in the vertical direction	The mean amount of upper incisor in the vertical direction
U3-U3	The width of maxillary anterior arch	The distance of upper canine cusp to canine cusp
UⅤ-UⅤ	The width of maxillary middle arch	The distance of both side second deciduous molar central fossa
U6-U6	The width of maxillary posterior arch	The distance of both side first molar central fossa
AL	The dental arch length	The perpendicular distance from the mesial point of the two upper incisor to the line connecting left-right first molar central fossa

### The method of statistical analysis

The SPSS 17.0 for Windows software was used for statistical analysis. The mean values and standard deviations (SD) were calculated for all the measurements. The paired t-test was used for comparisons between T1 and T2 data. The independent t-test was used for comparisons between T2 and control data. Significance was determined at a level of P <0.05.

All measurements were repeated after one month to assess intra-rater reliability. The statistical differences between the repeated measurements and originals were evaluated by paired t-test. The method error was calculated as: s = ∑(d)2/2n (d is deviation between the two measurements; n is the number of paired double measurements) [27].

## Results

No statistical differences were found between the repeated and original measurements (P = 0.891). The age distribution between the patients of T2 group and control group showed no significant difference (P = 0.803). This study’s method errors were 0.16 to 0.21 mm for the 3D linear measurement, 3.56 to 4.89 mm^2^ for the area measurement, and 6.33 to 7.21 mm^3^ for the volume measurement.

Comparing with the data from T1 group, the subspinale (A) of T2 group was advanced by 2.88±0.88mm in the horizontal direction and 1.23±1.32 mm in the vertical direction. The Upper incisor (UI) of T2 group was moved anterosuperiorly by 3.28±1.05 mm and 1.49±1.06mm in horizontal and vertical direction, respectively. The gnathion (Gn) of T2 group was moved posteroinferiorly by 3.40±0.93 mm and 3.66±1.06 mm in horizontal and vertical direction, respectively. The hyoid bone of T2 group was moved in posterior and inferior directions. After the PE treatment, a significant increase occurred in the dental arch length and width (Table 3). The upper airway showed a significant increase in the volume and mean cross-sectional area at the nasopharynx, velopharynx and glossopharynx regions, but the hypopharynx showed no significant difference. The AP/LR ratio of the velopharynx and glossopharynx was increased, but the AP/LR ratio of the hypopharynx was decreased (Table 4).

**Table 3:** The measured variables of hard tissues in PE group and control group(mm).

	Control(n = 30)		PE(n = 30)				P	
			T1		T2			
Variables	Mean	SD	Mean	SD	Mean	SD	T1&T2	T2&control
U3-U3	32.84	3.61	32.11	3.20	34.51	3.13	.000	.002
UⅤ-UⅤ	44.12	1.98	43.41	2.14	45.66	2.33	.000	.012
U6-U6	47.91	2.36	47.15	2.54	49.40	2.81	.000	.004
AL	29.11	2.12	28.86	2.84	31.41	3.21	.000	.001
C3-H	29.13	3.28	29.02	2.90	28.09	3.24	.020	.017
C3Me-H	2.89	1.59	2.52	1.64	3.31	2.26	.041	.043

**Table 4:** The measured variables of airway in control group and PE group.

	Control(n = 30)		PE(n = 30)				P	
			T1		T2			
Variables	mean	SD	mean	SD	mean	SD	T1&T2	T2&control
NA-V(mm^3^)	39103.31	809.84	3862.38	929.09	4387.60	972.43	.006	.021
VE-V(mm^3^)	5398.16	857.39	4896.03	716.54	5894.18	759.28	.001	.028
GL-V(mm^3^)	2956.32	410.33	2490.48	461.43	2849.30	481.79	.016	.074
HY-V(mm^3^)	2283.89	389.75	2269.24	494.09	2129.76	598.67	.107	.112
UA-V(mm^3^)	16397.39	798.34	15978.15	1490.00	17454.12	1467.50	.001	.032
NA-mCSA (mm^2^)	308.23	50.09	309.57	92.13	341.89	108.53	.031	.032
VE-mCSA (mm^2^)	264.11	39.25	257.22	43.39	287.21	41.73	.016	.041
GL-mCSA (mm^2^)	189.32	30.84	179.31	27.32	188.04	30.70	.042	.357
HY-mCSA (mm^2^)	218.01	32.57	216.96	47.36	198.82	37.59	.094	.102
NA-AP/LR	0.79	0.12	0.81	0.17	0.76	0.11	.112	.131
VE-AP/LR	0.81	0.09	0.78	0.27	0.99	0.19	.002	.008
GL-AP/LR	0.51	0.14	0.48	0.13	0.50	0.15	.045	.121
HY-AP/LR	0.46	0.10	0.45	0.14	0.41	0.10	.041	.038

As for the comparison of the control group and T2 group, the length and width of the maxillary dental arch showed significant increase after the PE treatment. The displacement of hyoid bone in the T2 group was more posterior. There was an increase in the volume of nasopharynx and velopharynx after the treatment, but no significant changes in the glossopharynx and hypopharynx were found following treatment. The AP/LR ratio of the velopharynx in the T2 group increased significantly, but the AP/LR ratio of the hypopharynx decreased (Table 4).

## Discussion

In this study, we evaluated the effects of PE treatment on the upper airway morphology compared with the control group through 3D reconstructive assessment. After PE treatment, the volume and cross-sectional area of nasopharynx, velopharynx and the total airway was increased. In addition, the shape of the velopharynx became more circular, and the shape of the hypopharynx became more elliptic. These data suggested that the PE treatment significantly changes the shape and size of upper airway.

The changes of the nasopharynx and oropharynx demonstrated that the upper airway was closely related to the changes of maxillary, mandible, hyoid and soft surrounding tissue. The orthopedic force of the protraction appliance may stimulate cellular activity in circum maxillary sutures, and facilitate the maxillary to move in a forward direction [7,28]. When the maxillary protraction is combined with rapid expansion, the maxillary bone is broadened and the suture between maxillary is opened by the rapid expansion [29]. Our study showed that changes of the width and length of the maxillary bone expanded the volume of the nasopharynx. Because of the increase of the sagittal diameter and the left-right diameter of nasopharynx, the shape of the nasopharynx region did not change significantly. This result is consistent with most previous studies [7–9]. The significant changes of the volume and shape of the velopharynx might be caused by the effects of PE treatment on the soft palate. After the PE treatment, the enlarged oral cavity provided a more large space for the tongue, but the mandible moved in a backward and downward direction and rotated in a clockwise direction. This displacement may limit the position of the tongue [30].Thus, there were no significant changes of the volume and shape of the glossopharynx. However, the significant changes of the glossopharynx on volume and shape in the PE group at T2 could be the result of growth, and not the PE treatment. Kilinç et al. [31] analyzed the effects of the sagittal pharyngeal dimensions of protraction and rapid palatal expansion in Class III malocclusion subjects, and found that the treatment improved the nasopharyngeal and oropharyngeal airway dimensions in the short term. However, Sayınsu et al.[32] found that the treatment improved nasopharyngeal but not oropharyngeal airway dimensions. The discrepancy of these results might be caused by inadequate clear demarcation of the oropharynx. Previous studies indicated that the skeletal structure of craniomaxillofacial and the soft tissues of the airway are the important factors to determine the morphology of upper airway [33–34]. The morphology and dimension of the oropharynx was affected by pharyngeal wall, soft palate and the soft tissues of tongue [35]. As the soft palate and tongue are the two independent soft tissues around the oropharynx, they might have different changes when suffering the force. Therefore, the detailed demarcation of the oropharynx is essential for the accurate evaluation of the upper airway. In this study, the oropharynx was divided into 2 parts: the velopharynx and the glossopharynx. Each part of oropharynx has its own components and surrounding structures. The velopharynx and glossopharynx were limited mostly by the soft palate and the tongue, respectively. Therefore, the nasopharynx and oropharynx of the upper airway had significant changes after the treatment, but the changes of the oropharynx were mainly on the velopharynx region.

The volume of the hypopharynx showed no significant changes after the PE treatment, but the shape of the hypopharynx became more elliptical, indicating that the morphology of the hypopharynx was affected by multiple factors. As the mandible moved backward and downward, the hyoid bone showed a posterior-inferior displacement. The muscles around tongue were dragged by the hyoid bone [36]. These changes of the hyoid bone and the muscles around tongue made a decreasing tendency of the volume of hypopharynx [21,37]. However, Li et al. [38] discovered that with increasing age, the size of upper airway in both children and teenagers increased, indicating that the normal growth played a vital role in the increasing volume of hypopharynx. Previous studies [10] indicated that the modifications in the sagittal airway dimensions induced by therapy or physiological growth showed great inter-individual variability in subjects with Class III malocclusion. Thus, the volume of hypopharynx showed no statistically significance, which might be a result of the great inter-individual variability, PE treatment and growth development during the treatment. Since the position of mandible and hyoid had a backward and downward displacement, which decreased the sagittal diameter of the hypopharynx, the shape of hypopharynx become more elliptical [7,8]. Therefore, the changes of the mandible, hyoid, great inter-individual variability and growth development were essential to the volume and shape of the hypopharynx [19,39,40].

The increase of nasopharynx and velopharynx after PE treatment is important for improving the ventilation function of the Class III malocclusion patients with maxillary skeletal deficiency. Li et al. [38] reported that the volume of nasopharynx in Chinese children with normal upper airway was 4177.52±2260.84mm^3^ in male and 3866.11±1651.64 mm^3^ in female. In addition, the volume of velopharynx in Chinese Han children with normal upper airway was 5756.4 ± 2277.1 mm^3^ in male and 6158.9 ± 2121.8 mm^3^ in female [41]. No significant difference in airway volume was found between the two genders. In our study, the volume of nasopharynx (3862.38±929.09mm^3^) and velopharynx (4896.03±716.54mm^3^) before PE treatment were smaller than the measurements of the normal upper airway from previous reports. Although the age of patients in our study was younger than normal children in previous studies, these Class III malocclusion patients with maxillary skeletal deficiency might have narrow nasopharynx and velopharynx. After PE treatment, the volume of nasopharynx (4387.60±972.43mm^3^) and the volume of velopharynx (5894.18±759.28mm^3^) were enlarged and became comparable to the previous measurements. Therefore, the PE treatment could expand nasopharynx and velopharynx, overcome the insufficiency, and improve the ventilation function of class III malocclusion patients. As the methods, demarcation of upper airway, subjects of our study were different from the previous studies, the comparison may not be accurate [38,41–43]. Therefore, the more detailed study of normal upper airway with specific age group is needed for evaluating the effects of PE treatment for Class III malocclusion patients with maxillary skeletal deficiency.

This study is limited due to the absence of long-term study and functional study. As the patients were still in an active growth period at the end of the PE treatment, their upper airway might have growth potential before craniofacial growth is completed. The change of morphology is one of the important factors for the upper airway function. The functional study of upper airway may involve other physiological effects. Therefore, the long-term and functional research is essential for the future studies.

## Conclusion

Compared to the untreated patients, the volume of nasopharynx and velopharynx of growing patients with Class III malocclusion and maxillary skeletal deficiency increases after the PE treatment. The volume of glossopharynx and hypopharynx remains unchanged in a short-term. In addition, the velopharynx becomes more circular and the hypopharynx becomes more elliptic in transverse shape after PE treatment.
